# Water and Air

**Published:** 1903-09-05

**Authors:** J. P. Sandlands


### EDITOR'S LETTER-BOX.

### "WATER AND AIR."

The Rev. J. P. Sandlands writes:—"Will you kindly permit me to make a few remarks in connection with your review of the above? 1. I owe you many thanks for it, and these I hasten to give. 2 Science is not eccentric, seeing it deals with facts; this is true, whether the science be much or little. 3. Ideas are chimeras if they are not represented by facts. 4. T. P. S. does not deal in paradoxes. 5. Many of those who seek and follow my advice live absolutely without liquids of any kind. 6. No man should follow rules. 7. The true man should be guided by principles. 8. I do not know that I have erred in any of these particulars."

